# The prevalence of platelet activating factor acetylhydrolase single nucleotide polymorphisms in relationship to necrotizing enterocolitis in Northwest Louisiana infants

**DOI:** 10.1186/2193-1801-2-294

**Published:** 2013-07-02

**Authors:** Senthilkumar Sankararaman, Krishna Yanamandra, Dawn Napper, Gloria Caldito, Ramasubbareddy Dhanireddy

**Affiliations:** Department of Pediatrics, Louisiana State University Health Sciences Center, Shreveport, LA 71130 USA; Department of Biometry, Louisiana State University Health Sciences Center, Shreveport, LA 71130 USA; Department of Pediatrics (Neonatology division), University of Tennessee Health Science Center, Memphis, TN 38163 USA

**Keywords:** Necrotizing enterocolitis, Neonates, Preterm, Single nucleotide polymorphisms, Platelet activating factor, PAF, PAF acetylhydrolase, PAFAH

## Abstract

**Purpose:**

Studies documented that platelet activating factor (PAF) and the enzyme platelet activating factor acetylhydrolase (PAFAH) play a very important role in the pathogenesis of neonatal necrotizing enterocolitis (NEC). In this retrospective, case-controlled pilot study, the authors investigated the prevalence of single nucleotide polymorphisms (Ile198Thr and Ala379Val) of the PAFAH gene.

**Subjects and methods:**

We screened 570 blood samples from both Caucasian and African-American preterm infants in the Northwest Louisiana population for the above mentioned PAFAH gene polymorphisms. Out of 570 infants, 36 had stage I or II NEC based on diagnostic coding, the International Classification of Diseases, 9th revision, Clinical Modification, 2009 (ICD-9-CM). The remaining infants without an ICD-9-CM diagnosis of NEC were recruited as control population. The DNA was isolated and restriction fragment length polymorphism microplate polymerase chain reaction assay was performed.

**Results:**

Variants of the PAFAH gene polymorphism (Ile198Thr and Ala379Val) frequencies were not significantly different between the infants with NEC and the control group (*P* value of 0.26 by either multiple logistic regression analysis or the Cochran-Mantel-Haenszel test).

**Conclusions:**

This is the first study of its kind in exploring the relationship between NEC and single nucleotide polymorphisms in the coding genes of the enzyme PAFAH. Our preliminary data demonstrated that adjusted for the effect of race, PAFAH polymorphisms (Ile198Thr and Ala379Val) have no significant effect on NEC.

## Background

Necrotizing enterocolitis (NEC) is the most common surgical gastrointestinal emergency in neonates with high morbidity and mortality. The exact etiology of NEC is still unknown and prematurity is considered as the most important risk factor Beeby & Jeffery (1992). More than 90% of the infants with NEC are born before 36 weeks gestation Henry & Moss (2009). Immaturity and ischemia of the gut, infection and introduction of hyperosmolar feeds are the key factors postulated in the development of NEC Henry & Moss (2009). Regardless of the underlying risk factors, the endogenous inflammatory mediators such as interleukins, platelet activating factor (PAF) and nitric oxide (NO) have been implicated in the final common pathogenesis of NEC Edelson et al. (1999; Ewer 2002; Muguruma et al. 1997; Caplan et al. 2005). PAF (1-O-alkyl-2-acetyl-sn-glycero-3-phosphocholine) is a potent pro-inflammatory mediator, involved in the signaling and activation of proinflammatory cells such as platelets, neutrophils, and macrophages Prescott et al. (1990). PAF causes a variety of biological functions such as epithelial apoptosis, increased vascular permeability, bronchoconstriction and smooth muscle contraction Hsueh et al. (1994; Caplan et al. 1992; Sun et al. 1994).

The proinflammatory effects of PAF are terminated by the enzyme PAF acetylhydrolase (PAFAH). It exists in two forms - intracellular and secretory. The secretory form is found in breast milk and in other body fluids Moya et al. (1994). Studies in human infants with NEC have shown increased luminal and systemic PAF and reduced expression of PAFAH Caplan et al. (1990). In animal models with NEC, increased levels of serum PAF and decreased serum levels of the PAFAH have also been clearly documented Lu et al. (2010). The role of PAF and PAFAH has been extensively documented in various disease states involving inflammation Tjoelker & Stafforini (2000).

Asthma and atopy are the prototypic diseases where the role of PAF mediating inflammation been extensively studied. Increased PAF and reduced PAFAH activity have been proven in asthma patients Tsukioka et al. (1996; Miwa et al. 1988). Ile198Thr (in exon 7, position 593;T > C) and Ala379Val (in exon 11, position 1136;T > C) variants in PAFAH gene have been associated with an increased risk for the development of atopy and asthma in Caucasian population Kruse et al. (2000). Even though the role of PAF in NEC is well documented, no prior study has been reported about the prevalence of PAFAH variants in NEC infants and the role of potential pathogenesis. The rationale for the present study is based on the research by Kruse et al. (2000). In neonates, the polymorphisms of coding genes of various enzymes involved in the inflammatory pathways can influence various conditions such as respiratory distress syndrome, bronchopulmonary dysplasia, intraventricular hemorrhage and retinopathy of prematurity. For example, in our previous studies, we have reported associations between genotypes of the nitric oxide synthase gene and two neonatal conditions namely intraventricular hemorrhage and retinopathy of prematurity Yanamandra et al. (2010; Vannemreddy et al. 2009). In this prototype study, we attempted to find the prevalence of the PAFAH variants (Ile198Thr and Ala379Val) in Northwest Louisiana infants both with and without NEC and any causal relationship between these variants and the NEC.

## Materials and Methods

### Subjects

Institutional review board (IRB) approval was obtained. We screened peripheral EDTA blood samples from 570 preterm infants admitted to the neonatal intensive care unit (NICU), Louisiana State University Health Sciences Center, Shreveport, Louisiana for the presence of Ile198Thr and Ala379Val polymorphisms.

The diagnosis of NEC was based on diagnostic coding, the International Classification of Diseases, 9th revision, Clinical Modification, 2009; ICD-9-CM code for the perinatal disorders of the digestive system – 777.5 is NEC in newborn; 777.50 is NEC in newborn unspecified; 777.51 is stage I NEC; 777.52 is stage II NEC (NEC with pneumatosis, without perforation) and 777.53 is stage III (NEC with perforation or NEC with pneumatosis and perforation) (The International Classification of Diseases, 9th revision, Clinical Modification, 2009).

Only limited clinical information was available to us such as ethnicity of the infants, gestational age and birth weight. The important clinical information pertinent to NEC such as feeding history, sepsis, presence of patent ductus arteriosus, use of inotropes, administration of indomethacin, radiological findings and surgical management were not available for analysis.

Out of 570 infants, 36 infants (20 Caucasian infants and 16 African-American infants) had either stage I or II NEC. The number of infants with stage I and II were equal in both ethnicities. In view of small numbers, stages I and II were combined into one category. The 36 infants in the NEC population did not have the ICD-9-CM diagnostic codes 777.5, 777.50 or 777.53. Also in this sample of 570 infants, no infants died. The remaining 534 infants (570–36) did not have any diagnostic coding for NEC, and they were recruited as the control population. The control population included 192 Caucasian and 342 African-American infants. The demographic and the gestational characteristics such as birth weight and gestational age for the NEC and the control groups were summarized in Table 1. In the Caucasian group, the birth weight and the gestational age in the NEC group and the control group were comparable (p value >0.05 by two sided z test). In the African-American group, the NEC population was significantly younger and more premature when compared to the control group (p value < 0.05 by two sided z test).

**Table 1:** **Summary statistics on demographic and gestational characteristics of the study subjects**

NEC	Ethnicity	N	Birth weight (gms)		Gestational age (weeks)	
			Range	Mean	Range	Mean
Yes	C	20	1100-2837	2149	29.7 - 36.8	34.2
No	C	192	720 - 2609	2278	27.6 - 36.8	33.9
Yes	AA	16	810 - 1510	1224	24.7 - 32.1	29.1
No	AA	342	540 - 2480	1965	23.4 - 36.8	32.8

NEC – necrotizing enterocolitis; C- Caucasian; AA – African-American.

### Methods

DNA was isolated from patients and controls using Qiagen blood mini DNA isolation kits. Microplate PCR method was carried out for each polymorphism. The genotype assay used was modified from the assay used by Kruse et al. (2000).

### Ala379Val polymorphism

Briefly 10 microlitre (μl) of the PCR mix contained the following components in the final concentration of dH20 5.35 μl, 25 mM MgCl2 1.3 μl, 10× Applied Biosystems buffer 1 μl, 3.75 pmoles of each of 4 dNTPs, forward and reverse PAFAH primers 0.5 μl, Taq polymerase (gold, Perkin-Elmer) 0.5 units, and 1–2 ng of patient DNA. The primer sequences used were: GGG AGA CAT AGA TTC AAC TG (forward) and CGT TTT GTA AGA ATG CTA ATG A (reverse). The PCR conditions used were : Initial denaturation for 10 min at 94°C, 35 cycles of the following program: 94°C for 45 seconds, 53°C for 90 seconds, 72°C for 90 seconds with the final extension at 72°C for 10 minutes.

Enzyme digestion was carried out using nuclear enzyme buffer (0.5 μl), bovine serum albumin (0.01 μl) and restriction endonuclease (Pst I). To 5 μl of the PCR product, 1 μl of the enzyme mix was added and incubated at 37°2C for 2 hours. The digested PCR fragments were separated by electrophoresis on 3% agarose gel for 2 hours. Ethidium bromide staining was used to reveal the fragments under an ultraviolet light transillumination.

### Ile198Thr polymorphism

Assay parameters and conditions were similar as in the Ala379Val except that the restriction enzyme was Pvu II instead of Pst I, and the primer sequences were as follows: ATA AAT AAT TTT TGC TTG TAT (forward) and AGA GCC AAG ACT TGT CAG CT (reverse).

## Results

### Distribution of PAFAH (Ile198Thr & Ala379Val) variants

In view of small sample size, both variants Ile198Thr and Ala379Val were combined for analysis. In Table 2, the study population was cross classified by three variables (ethnicity, presence or absence of NEC and the study genotypes).

NEC group: Among the NEC group, the homozygous abnormal genotype (198ThrThr + 379ValVal) was not seen in any infants. Eight infants (1 Caucasian and 7 African-American infants) had heterozygous abnormal genotype (198IleThr + 379AlaVal). The normal homozygous (198IleIle + 379AlaAla) genotype was found in 28 infants (19 Caucasian and 9 African-American infants).

Control group: Among the control population of healthy infants without NEC, homozygous abnormal genotype (198ThrThr + 379ValVal) was seen in 29 infants (7 Caucasian + 22 African-American infants). Heterozygous abnormal genotype (198IleThr + 379AlaVal) was identified in 159 infants (36 Caucasian + 123 African-American infants). Normal homozygous (198IleIle + 379AlaAla) genotype was found in 346 infants (149 Caucasian + 197 African-American infants).

**Table 2:** **Study population classified based on three variables (ethnicity, presence or absence of NEC and the study genotypes)**

		PAFAH Genotypes			
Ethnicity	NEC	(1)	(2)	(3)	Total
Caucasian	Yes	0	1	19	20
Caucasian	No	7	36	149	192
African-American	Yes	0	7	9	16
African-American	No	22	123	197	342
Total		29	167	374	570

PAFAH Genotypes – (1) is 198 ThrThr + 379 ValVal (homozygous abnormal genotype), (2) is 198 IleThr + 379AlaVal (heterozygous abnormal genotype) and (3) is 198IleIle + 379AlaAla (homozygous normal genotype).

### Statistical analysis

Statistical analysis was performed using SAS Version 9.2 (SAS Institute Inc., Cary, NC, USA). A total of 570 infants were classified into three PAFAH genotypes-198ThrThr + 379ValVal or homozygous abnormal genotype (represented as genotype 1), 198 IleThr + 379AlaVal or heterozygous abnormal genotype (represented as genotype 2) and 198IleIle + 379AlaAla or homozygous normal genotype (represented as genotype 3). Table 2 shows the frequency of the genotypes stratified by ethnicity (Caucasian or African-American) and disease status (NEC present or absent). Given the relatively small sample size for genotype 1, which has zero infants with occurrence of NEC, it was combined with genotype 2 and the combined group was considered abnormal genotype. This combined cross classified table is illustrated in Table 3.

**Table 3:** **Study population cross classified by three variables (ethnicity, presence or absence of NEC and the study genotypes)**

Ethnicity	NEC	Abnormal genotype	Normal genotype	Total
Caucasian	Yes	1	19	20
Caucasian	No	43	149	192
African-American	Yes	7	9	16
African-American	No	145	197	342
Total		196	374	570

In view of small numbers, the homozygous abnormal genotype (genotype 1 in Table 2) and heterozygous abnormal genotype (genotype 2 in Table 2) were combined into one category (abnormal genotype).

Multiple logistic regression analysis was used to determine the effects of genotype and race on NEC occurrence adjusted for the effects of each other with the following results (Table 4) Adjusted for the effect of genotype, odds for NEC among Caucasian infants were 2.03 times the odds for NEC among African-American infants in the study population. The adjusted odds ratio (OR) was significant with p-value <0.05 and a 95% confidence interval (CI), which did not contain 1 (value of OR under the null hypothesis of no association between race and NEC).

**Table 4:** **Results of multiple logistic regression analysis on the study population for the occurrence of NEC**

Factor	Odds ratio (OR)	95% CI for OR	p-value
Race (C vs. AA)	2.03	1.01 – 4.07	0.046*
Genotype			
Abnormal vs. Normal	0.62	0.27 – 1.42	0.26^NS^

C- Caucasian; AA – African-American. *Significant at 5% level (0.01 < p-value < 0.05) ^NS^ Not significant at 5% level (p-value > 0.05).

Adjusted for effect of race, genotype had no significant effect on NEC; that is, adjusted for race, odds for NEC among infants with abnormal genotype was similar to those among infants with normal genotype. [Note: Although the adjusted OR for genotype was not significant, the calculated OR (< 1) seemed to suggest that infants with abnormal genotype had smaller odds for NEC than the infants with normal genotype].

Alternatively, since the focus of the study was on the role of polymorphisms in the etiology of NEC, the 3-way cross classified table was analyzed with the 2 races as strata in determining the association between PAFAH genotype and NEC using the Cochran-Mantel-Haenszel (CMH) test.

The CMH statistic, its 95% CI and its p-value were calculated as CMH statistic = 1.65, 95% CI = (0.71 to 3.84), p-value = 0.26. The CMH statistic was not significant as its 95% CI contained 1 and its p-value > 0.05. Thus, when the infants were stratified by race (Caucasian or African-American) there was no association between genotype and NEC. The p-value for the CMH statistic (p = 0.26) was the same as the p-value for the adjusted OR for abnormal vs. normal genotype (p = 0.26) using multiple logistic regression analysis. Also, the CMH did not test for effect of race; rather it was used to stratify the infants.

## Discussion

Necrotizing enterocolitis (NEC) remains one of the leading causes of morbidity and mortality in neonatal intensive care units, with an incidence of 10% among very low birth weight infants (<1500 grams) Uauy et al. (1991) and a mortality of approximately 26% Hack et al. (1991). The lower the gestational age and birth weight, higher is the risk of developing NEC. Despite improvements in overall neonatal survival, this mortality rate has not changed significantly over the last three decades Henry & Moss (2009; Ewer 2002). In addition to the mortality, NEC is associated with high morbidity resulting from multiple surgeries, need for parenteral nutrition, cholestatic liver disease and development of short gut syndrome.

Although several predisposing factors such as prematurity, bacterial infection, enteral feeding and gut ischemia have been identified, the exact pathogenesis of NEC remains unknown Beeby & Jeffery (1992). Regardless of the initial triggering factor, the final pathology is similar which strengthens the possibility of the role of inflammatory mediators such as platelet activating factor (PAF) Ewer (2002; Muguruma et al. 1997; Caplan et al. 2005).

PAF is an endogenous phospholipid with potent proinflammatory actions. It is synthesized from the plasma membrane precursors by cells such as intestinal cells and neutrophils. The half-life is very short and it is degraded by the enzyme PAF acetylhydrolase (PAFAH). PAFAH can be therefore be considered as the functional antagonist of PAF. The PAF receptor is a G protein-coupled seven transmembrane domain receptor family (GPCR). PAF combines with its receptor which is present on most cells resulting in activation of the inflammatory cascade Caplan et al. (2005; Prescott et al. 1990). The exact mechanism of pathogenesis of PAF in NEC is not known, but exposure to a high local concentration of PAF results in increased mucosal permeability, apoptosis, activation of inflammatory cascade and finally bowel necrosis Frost et al. (2008).

Numerous clinical and experimental studies have substantially strengthened our understanding of the role of PAF and PAFAH in the pathogenesis of NEC. The PAFAH activity is generally lower in newborns compared to adults, which enhances the susceptibility of neonates to the NEC Caplan et al. (1990). NEC induced by intravenous administration of PAF has been documented in neonatal experimental piglet models Ewer et al. (2004) and also in rats Gonzalez-Crussi & Hsueh (1983). Both in human and animal subjects, it has been proven that in NEC, the local as well as systemic concentrations of PAF were significantly high with concurrent decreased expression of PAFAH Caplan et al. (1990; Lu et al. 2010). Increased concentration of stool PAF in NEC has also been documented Amer et al. (2004). In contrast, reduced activity and concentration of PAFAH has been found in the neonatal population particularly in infants with NEC Caplan et al. (1990). Additionally, enteral administration of recombinant PAFAH or PAF receptor antagonist to neonatal rats resulted in a significant decrease in NEC Caplan et al. (1997;1997) strengthening the concept of functional antagonism of PAFAH in the NEC medicated by PAF.

The human PAF acetylhydrolase gene is located in chromosome 6p12.p21.1. Stafforini et al. (1996). Reduced PAFAH has been documented in many disease states where PAF plays a role in mediating the inflammation such as in asthma Kruse et al. (2000), sepsis Graham et al. (1994), stroke Satoh (2008; Hiramoto et al. 1997), multiple organ failure Partrick et al. (1997) and myocardial infarction Yamada et al. (1998). A difference in PAFAH activity between individuals/ethnicities has been extensively studied and genetic variations in the candidate genes has been proposed as the etiology for this difference in activity Tjoelker & Stafforini (2000; Stafforini et al. 1996). The single nucleotide polymorphisms (SNPs) commonly described in the human PAFAH gene were Arg92His (exon 4), Ile198Thr (exon 7), Val279Phe (exon 9), Gln281Arg (exon 9) and Ala379Val (exon 11) Li et al. (2009). The influence of these PAFAH polymorphisms is well studied in many disease states where PAF plays a significant role in the pathogenesis. The role of SNPs in the pathogenesis of diseases is variable in different ethnicities and controversial Kruse et al. (2000; Li et al. 2009). For example, in the Japanese population, two loss of function mutations on PAFAH gene have been described in asthmatic children and similar mutations were not seen in the Caucasian population with asthma Kruse et al. (2000; Stafforini et al. 1999).

The two PAFAH variants (Ile198Thr and Ala379Val) were also studied in conditions such as asthma Kruse et al. (2000), aging Campo et al. (2008), acute respiratory distress syndrome Li et al. (2009) and in schizophrenia Bell et al. (1997; Ohtsuki et al. 2002). Even though the role of PAF in NEC is well known, similar SNP studies in the PAFAH gene have not been reported in the literature. We selected the two PAFAH variants (Ile198Thr and Ala379Val) based on the study by Kruse et al. (2000). Kruse et al. (2000) demonstrated that in Caucasian population, Thr198 and Val379, the two common variants of the PAFAH gene were associated with lower substrate affinities (higher *Km*) of PAFAH. They also demonstrated that decreased affinity of PAFAH for PAF results in reduced catalytic activity of PAFAH, which is believed to further prolong the activity of PAF and increase the risk of development of asthma and atopy. We applied this rationale to our study and attempted to find out the relationship between the two PAFAH variants and NEC.

In our study population, adjusted for the effect of race, genotypic variants had no significant effect on NEC; that is, adjusted for race, odds for NEC among infants with an abnormal genotype was similar to those among infants with a normal genotype. Because of the controversial relationship of variant genotypes to the PAFAH enzyme levels in different ethnicities Kruse et al. (2000; Stafforini et al. 1999) and our study being the first study of its kind in the literature, the exact significance of our observations is unknown at present.

There are several limitations in this study that warrant discussion. First, the sample size was small. The concurrent measurements of PAF and PAFAH activity would be more meaningful. As our study was retrospective, concurrent measurements of PAF and PAFAH levels were not feasible. Another limitation is that the definition and the inclusion criteria for NEC was strictly based on diagnostic coding, the International Classification of Diseases, 9th revision, Clinical Modification, 2009; ICD-9-CM. Even though very desirable, pertinent clinical information such as feeding history, sepsis, presence of patent ductus arteriosus, use of inotropes, indomethacin, etc. which play a vital role in the etio-pathogenesis of NEC were not taken for analysis due to unavailability. Our study sample did not have advanced stages of NEC such as stage 3 NEC (NEC with intestinal perforation or NEC with pneumatosis with perforation) or infants who died from NEC which is also a limitation.

Despite the limitations, our study is a prototype pilot study in providing the prevalence of PAFAH gene polymorphisms in the Northwest Louisiana population. Also we attempted to find a relationship between PAFAH variants and NEC. A Medline search using the terms “necrotizing enterocolitis” and “platelet activating factor acetylhydrolase” revealed 20 articles. To the best of our knowledge, a detailed review of these articles did not reveal any previous study showing the role of PAFAH polymorphisms in the pathogenesis of NEC. Based on our results, we conclude that PAFAH genotype variants (Ile198Thr and Ala379Val) have no significant effect on NEC. A prospective study involving a larger population with concurrent measurements of PAF and PAFAH along with detailed clinical information is recommended to confirm or refute our initial findings.

## References

[CR1] Amer MD, Hedlund E, Rochester J (2004). Platelet-activating factor concentration in the stool of human newborns: effects of enteral feeding and neonatal necrotizing enterocolitis. Biol Neonate.

[CR2] Beeby PJ, Jeffery H (1992). Risk factors for necrotising enterocolitis: the influence of gestational age. Arch Dis Child.

[CR3] Bell R, Collier DA, Rice SQ (1997). Systematic screening of the LDL-PLA2 gene for polymorphic variants and case control analysis in schizophrenia. Biochem Biophys Res Commun.

[CR4] Campo S, Sardo MA, Trimarchi G (2008). Platelet activating factor-acetylhydrolase (PAF-AH) activity and HDL levels, but not PAF-AH gene polymorphisms, are associated with successful aging in Sicilian octogenarians. Aging Clin Exp Res.

[CR5] Caplan MS, Hsueh W, Kelly A (1990). Serum PAF acetylhydrolase increases during neonatal maturation. Prostaglandins.

[CR6] Caplan MS, Kelly A, Hsueh W (1992). Endotoxin and hypoxia-induced intestinal necrosis in rats: the role of platelet activating factor. Pediatr Res.

[CR7] Caplan MS, Lickerman M, Adler L, Dietsch GN, Yu A (1997). The role of recombinant platelet-activating factor acetylhydrolase in a neonatal rat model of necrotizing enterocolitis. Pediatr Res.

[CR8] Caplan MS, Hedlund E, Adler L (1997). The platelet-activating factor receptor antagonist WEB 2170 prevents neonatal necrotizing enterocolitis in rats. J Pediatr Gastroenterol Nutr.

[CR9] Caplan MS, Simon D, Jilling T (2005). The role of PAF, TLR, and the inflammatory response in neonatal necrotizing enterocolitis. Semin Pediatr Surg.

[CR10] Edelson MB, Bagwell CE, Rozycki HJ (1999). Circulating pro- and counterinflammatory cytokine levels and severity in necrotizing enterocolitis. Pediatr.

[CR11] Ewer AK (2002). Role of platelet-activating factor in the pathophysiology of necrotizing enterocolitis. Acta Paediatr Suppl.

[CR12] Ewer AK, Al-Salti W, Coney AM (2004). The role of platelet activating factor in a neonatal piglet model of necrotising enterocolitis. Gut.

[CR13] Frost BL, Jilling T, Caplan MS (2008). The importance of pro-inflammatory signaling in neonatal necrotizing enterocolitis. Semin Perinatol.

[CR14] Gonzalez-Crussi F, Hsueh W (1983). Experimental model of ischemic bowel necrosis. The role of platelet-activating factor and endotoxin. Am J Pathol.

[CR15] Graham RM, Stephens CJ, Silvester W, Leong LL, Sturm MJ, Taylor RR (1994). Plasma degradation of platelet-activating factor in severely ill patients with clinical sepsis. Crit Care Med.

[CR16] Hack M, Horbar J, Malloy M (1991). Very low birth weight outcomes of the National Institute of Child Health and Human Development neonatal network. Pediatr.

[CR17] Henry MC, Moss RL (2009). Necrotizing enterocolitis. Annu Rev Med.

[CR18] Hiramoto M, Yoshida H, Imaizumi T, Yoshimizu N, Satoh K (1997). A mutation in plasma platelet-activating factor acetylhydrolase (Val279Phe) is a genetic risk factor for stroke. Stroke.

[CR19] Hsueh W, Caplan MS, Sun X (1994). Platelet-activating factor, tumor necrosis factor, hypoxia and necrotizing enterocolitis. Acta Paediatr Suppl.

[CR20] Kruse S, Mao XQ, Heinzmann A (2000). The Ile198Thr and Ala379Val variants of plasmatic PAF-Acetylhydrolase impair catalytical activities and are associated with atopy and asthma. Am J Hum Genet.

[CR21] Li S, Stuart L, Zhang Y (2009). Inter-individual variability of plasma PAFacetylhydrolase activity in ARDS patients and PAFAH genotype. J Clin Pharm Ther.

[CR22] Lu J, Pierce M, Franklin A (2010). Dual roles of endogenous platelet-activating factor acetylhydrolase in a murine model of necrotizing enterocolitis. Pediatr Res.

[CR23] Miwa M, Miyake T, Yamanaka T (1988). Characterization of serum platelet-activating factor (PAF) acetylhydrolase. Correlation between deficiency of serum PAF acetylhydrolase and respiratory symptoms in asthmatic children. J Clin Invest.

[CR24] Moya FR, Eguchi H, Zhao B (1994). Platelet-activating factor acetylhydrolase in term and preterm human milk: a preliminary report. J Pediatr Gastroenterol Nutr.

[CR25] Muguruma K, Gray PW, Tjoelker LW (1997). The central role of PAF in necrotizing enterocolitis development. Adv Exp Med Biol.

[CR26] Ohtsuki T, Watanabe H, Toru M, Arinami T (2002). Lack of evidence for associations between plasma platelet-activating factor acetylhydrolase deficiency and schizophrenia. Psychiatry Res.

[CR27] Partrick DA, Moore EE, Moore FA, Biffl WL, Barnett CC (1997). Reduced PAFacetylhydrolase activity is associated with postinjury multiple organ failure. Shock.

[CR28] Prescott SM, Zimmermann GA, McIntyre TM (1990). Platelet activating factor. J Biol Chem.

[CR29] Satoh K (2008). Plasma platelet-activating factor acetylhydrolase (PAF-AH) deficiency as a risk factor for stroke. Brain Nerve.

[CR30] Stafforini DM, Satoh K, Atkinson DL (1996). Platelet-activating factor acetylhydrolase deficiency. A missense mutation near the active site of an anti-inflammatory phospholipase. J Clin Invest.

[CR31] Stafforini DM, Numao T, Tsodikov A, Vaitkus D, Fukuda T, Watanbe N, Fueki N (1999). Deficiency of platelet activating factor acetylhydrolase is a severity factor for asthma. J Clin Invest.

[CR32] Sun X, Caplan MS, Hsueh W (1994). Tumour necrosis factor and endotoxin synergistically activate intestinal phospholipase A2 in mice. Role of endogenous platelet activating factor and effect of exogenous platelet activating factor. Gut.

[CR33] The International Classification of Diseases, 9th revision, Clinical Modification 2009. ICD-9-CM; perinatal disorders of the digestive system available at (accessed on June 12, 2013)

[CR34] Tjoelker LW, Stafforini DM (2000). Platelet-activating factor acetylhydrolases in health and disease. Biochim Biophys Acta.

[CR35] Tsukioka K, Matsuzaki M, Nakamata M (1996). Increased plasma level of plateletactivating factor (PAF) and decreased serum PAF acetylhydrolase (PAFAH) activity in adults with bronchial asthma. J Investig Allergol Clin Immunol.

[CR36] Uauy R, Fanaroff AA, Korones S (1991). Necrotizing enterocolitis in very low birth weight infants: biodemographic and clinical correlates. J Pediatr.

[CR37] Vannemreddy P, Notarianni C, Yanamandra K (2009). Is an endothelial nitric oxide synthase gene mutation a risk factor in the origin of intraventricular hemorrhage?. Neurosurg Focus.

[CR38] Yamada Y, Ichihara S, Fujimura T, Yokota M (1998). Identification of the G994 T missense in exon 9 of the plasma platelet-activating factor acetylhydrolase gene as an independent risk factor for coronary artery disease in Japanese men. Metabolism.

[CR39] Yanamandra K, Napper D, Pramanik A (2010). Endothelial nitric oxide synthase genotypes in the etiology of retinopathy of prematurity in premature infants. Ophthalmic Genet.

